# A network-based method for predicting disease-associated enhancers

**DOI:** 10.1371/journal.pone.0260432

**Published:** 2021-12-08

**Authors:** Duc-Hau Le

**Affiliations:** School of Computer Science and Engineering, Thuyloi University, Hanoi, Vietnam; University of Science and Technology Liaoning, CHINA

## Abstract

**Background:**

Enhancers regulate transcription of target genes, causing a change in expression level. Thus, the aberrant activity of enhancers can lead to diseases. To date, a large number of enhancers have been identified, yet a small portion of them have been found to be associated with diseases. This raises a pressing need to develop computational methods to predict associations between diseases and enhancers.

**Results:**

In this study, we assumed that enhancers sharing target genes could be associated with similar diseases to predict the association. Thus, we built an enhancer functional interaction network by connecting enhancers significantly sharing target genes, then developed a network diffusion method RWDisEnh, based on a random walk with restart algorithm, on networks of diseases and enhancers to globally measure the degree of the association between diseases and enhancers. RWDisEnh performed best when the disease similarities are integrated with the enhancer functional interaction network by known disease-enhancer associations in the form of a heterogeneous network of diseases and enhancers. It was also superior to another network diffusion method, i.e., PageRank with Priors, and a neighborhood-based one, i.e., MaxLink, which simply chooses the closest neighbors of known disease-associated enhancers. Finally, we showed that RWDisEnh could predict novel enhancers, which are either directly or indirectly associated with diseases.

**Conclusions:**

Taken together, RWDisEnh could be a potential method for predicting disease-enhancer associations.

## 1. Introduction

Enhancers are genomic cis-regulatory elements that activate transcription of their target genes, thus playing an important role in the pathogenesis of complex diseases. Indeed, genetic alterations of enhancers have been proven to contribute to disease progression [1]. Until now, more than three million enhancers have been identified by international consortiums such as ENCODE [2], FANTOM [3, 4], and NIH Epigenome Roadmap [5] using computational methods [6]. To accumulate functions of enhancers, annotation databases for enhancers have also been built. For example, EnhancerAtlas [7] is a resource for enhancer annotation and analysis in 105 human cell/tissue types. The target genes and their expression of enhancers are also integrated into GeneHancer [8] and McEnhancer [9], respectively.

Besides functions accumulated in the annotation databases, enhancers’ functions in terms of pathology are getting more focused. Indeed, genetic variants of enhancers play important roles in disease progression [1] because enhancers are regulatory elements that alter the expression level of their target genes. Mutations of enhancers can be associated with disease [10]. However, most studies about disease-enhancer associations are carried out for individual enhancers [11–13]. Recently, information on these associations from literature has been collected into a DiseaseEnhancer database and is publicly available [14]. However, only a small set of enhancers has been related to diseases. Therefore, there is a pressing need to predict novel disease-enhancer associations using computational methods.

In this study, we present a method RWDisEnh to predict novel disease-enhancer associations. The problem can be formulated as a ranking of candidate enhancers/diseases based on their relative importance to a disease/enhancer of interest, respectively. It was said that if an enhancer targets a disease-associated gene, then this enhancer is functionally connected to the disease [15]. Thus, we assumed that enhancers sharing target genes are associated with diseases that have similar phenotypes. Firstly, we built networks of enhancers and diseases based on functional interactions among enhancers and similarities among diseases as well as known disease-enhancer associations. The functional interaction between every pair of enhancers was assessed based on the significant sharing of their target genes to form a homogeneous network of enhancers (i.e., an enhancer functional interaction network where all nodes are enhancers). The similarity between every pair of diseases was calculated based on semantic similarity between two corresponding Disease Ontology (DO) terms [16] to form a homogeneous network of diseases (i.e., a disease similarity network where all nodes are diseases). The two homogeneous networks were then connected by known disease-enhancer associations collected from DiseaseEnhancer [14] to form a heterogeneous network of diseases and enhancers. Secondly, a random walk with restart (RWR) scheme on these networks was proposed to estimate the degree of association between a disease and an enhancer. RWR is the state-of-the-art guilt-by-association approach [17] and has been successfully used for various problems in biomedical research [18], especially ones in predicting disease-associated biomarkers such as genes [19–22] and non-coding RNAs [23–28]. Besides, RWR is also shown its dominance in other applications such as the prediction of drug-target interactions [29] and disease-related microRNA-environmental factor interactions [30]. In addition, RWR was proven to be the best one among network-based methods, including other commonly used network diffusion methods, proposed for the prediction of disease-gene associations [31]. To demonstrate the added value of the homogeneous networks, we compared the prediction performance of RWDisEnh on the heterogeneous network of diseases and enhancers with that on the enhancer functional interaction network and the disease similarity network. Experimental results show that RWDisEnh achieved the best performance in terms of AUC (area under the ROC curve) when it was performed on the heterogeneous network of diseases and enhancers.

To our knowledge, RWDisEnh is among the first network-based ones proposed for the prediction of disease-enhancer associations. As a kind of network diffusion method, RWR globally searches on the networks for novel enhancers/diseases associated with a disease/an enhancer of interest, respectively. To show the advance of RWDisEnh, we compared RWDisEnh with another network diffusion method, i.e., PageRank with Priors [32], on the enhancer functional interaction network. As assumed that enhancers sharing target genes are associated with similar diseases and defined a functional interaction between two enhancers using their shared target genes; thus, we additionally assessed whether enhancers neighboring with known disease-associated enhancers in the enhancer functional interaction network can be good candidates using MaxLink method [33,34]. In contrast to the network diffusion methods, MaxLink locally searches neighbors of known disease-associated enhancers for the novel ones. Experimental results showed that the prediction performance of the three methods is comparable on the enhancer functional interaction network; however, they were all worse than that of RWDisEnh on the disease similarity network and the heterogeneous network. Finally, we showed that RWDisEnh could predict novel enhancers associated with diseases with supporting direct and indirect evidence from genome-wide association studies and literature, respectively.

## 2. Materials and methods

### 2.1 RWDisEnh

**Fig 1:**
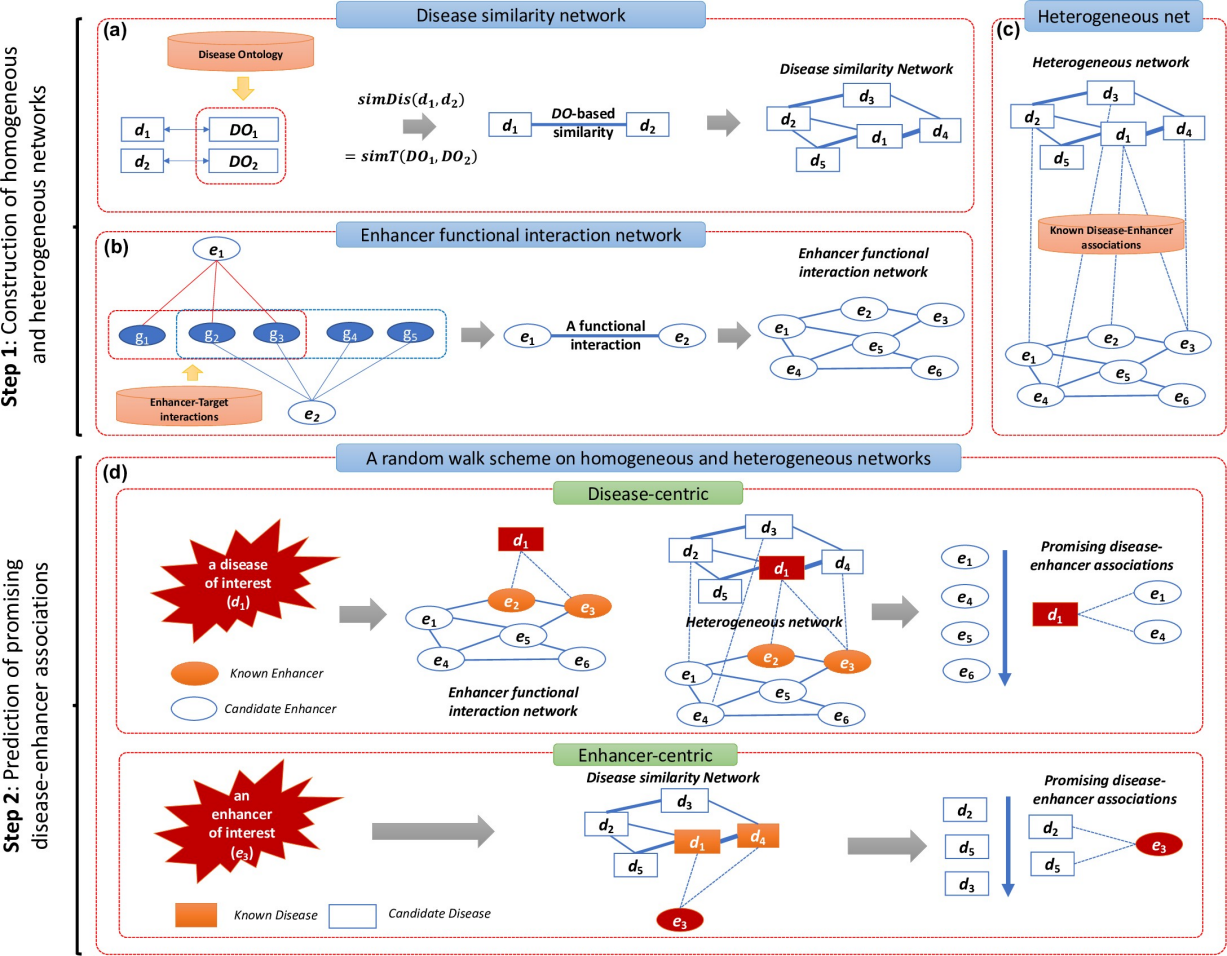
Illustration of RWDisEnh method. RWDisEnh includes two main steps. *Step 1—Construction of homogeneous and heterogeneous networks*: **(a)** A disease similarity network was formed based on DO-based similarities between every pair of mapped DO terms. **(b)** An enhancer functional interaction network was constructed by connecting every pair of enhancers significantly sharing target genes. **(c)** A heterogeneous network of diseases and enhancers was built by connecting the enhancer functional interaction network, the disease similarity network, and known disease-enhancer associations. *Step 2—Prediction of promising disease-enhancer associations*: **(d)** A random walk model was proposed on the networks to rank candidate enhancers/diseases. For disease-centric view: Given a disease *d*_1_, the goal is to rank all candidate enhancers (*e*_1_, *e*_4_, *e*_5_, and *e*_6_) by their relevance to *d*_1_. For enhancer-centric view: Given an enhancer *e*_3_, the goal is to rank all candidate diseases (*d*_2_, *d*_3_, and *d*_5_) by their relevance to *e*_3_. Finally, highly ranked candidates (e.g., *e*_1_, *e*_4_ for the disease-centric, and *d*_2_, *d*_5_ for the enhancer-centric) were selected as promising ones to be associated with the disease of interest (*d*_1_)/the enhancer of interest (*e*_3_), respectively.

In this section, we describe the RWDisEnh method. Briefly, first, we built an enhancer functional interaction network, a disease similarity network, and a heterogeneous network of diseases and enhancers. The disease similarity network was formed based on DO-based similarity between every pair of mapped DO terms (Fig 1(A)). The enhancer functional interaction network was constructed by connecting every pair of enhancers where their target genes are significantly overlapped (Fig 1(B)). Then, these two networks were connected using known disease-enhancer associations collected from DiseaseEnhancer [14] to construct the heterogeneous network of diseases and enhancers (Fig 1(C)). Finally, a random walk model was proposed to predict novel disease-enhancer associations based on the constructed networks (Fig 1(D)).

#### 2.1.1 Construction of a disease similarity network

To construct the DO-based disease similarity network, we calculated the similarity between any pair of mapped DO terms in the set of 2,161 DO terms having annotations in the DGA database [35]. Disease Ontology (DO) is a standardized structured vocabulary database for human disease to provide the biomedical community with consistent, reusable, and sustainable descriptions of human disease terms and related medical vocabulary disease concepts [16]. As with other biomedical ontologies [36], DO terms are organized as a directed acyclic graph where the term "disease" is defined as a root; meanwhile, other terms can be a leaf, a child, or a parent of others. DGA database provides a comprehensive and integrative annotation of the human genes by DO terms [35].

The similarity between two ontology terms was calculated based on the information content (*IC*) of each term, which is defined as the following:

IC(t)=−log(p(t)) (1)

where *p*(*t*) is the probability of term t occurring in a corpus (i.e., an annotation database, e.g., DGA for DO). More specifically, i.e., $p(t)=\frac{f(t)}{f(root)}$ such that *f*(*t*) = *Annot*(*t*)+∑_*c*∈*Children*(*t*)_*f*(*c*). In this formula, *Annot*(*t*) means the number of genes annotated with *t* in the corpus, and *Children*(*t*) represents the set of children terms of *t* in the DO graph. *root* is the root term of the DO graph. Then, the semantic similarity between the two DO terms, *t*_*i*_ and *t*_*j*_, based on the most informative common ancestor approach Resnik [37], is calculated as follows:

$$simTerm(t_{i},t_{j})=\underset{c\in P(t_{i},t_{j})}{\max}\left(IC(c)\right)$$ (2)

where *P*(*t*_*i*_, *t*_*j*_) is the set of shared ancestors of *t*_*i*_ and *t*_*j*_.

For a pair of diseases *d*_*i*_ and *d*_*j*_, which are directly mapped to *t*_*i*_ and *t*_*j*_, respectively, the similarity between them is defined as follows (Fig 1(A)):

$$w_{ij}=simDis(d_{i},d_{j})=simTerm(t_{i},t_{j})$$ (3)

We calculated the similarity for every pair of DO terms in a total of 2,152 DO terms to construct a DO-based disease similarity network, *G*_*D*_(*V*_*D*_, *E*_*D*_). By selecting pairs having *simDis*(*d*_*i*_, *d*_*j*_)>0, we also constructed a DO-based disease similarity network containing 806,505 interactions. Fig 1(A) illustrates the construction of the disease similarity network. This network was then represented as an adjacency matrix *W*_*D*_, where its element (*W*_*D*_)_*i*,*j*_ was set to *w*_*ij*_ representing the similarity between disease *d*_*i*_ and *d*_*j*_.

#### 2.1.2 Construction of an enhancer functional interaction network

It was assumed that if an enhancer targets a disease-associated gene, then this enhancer is functionally connected to the disease [15]. Thus, we here define a functional interaction between two enhancers using shared target genes. To this end, we collected known enhancer-target gene interactions between 792 enhancers and 667 target genes from the DiseaseEnhancer database [14]. Then, a functional interaction between two enhancers *e*_*i*_ and *e*_*j*_ was defined if there is significant overlap between their target sets using the hypergeometric distribution

$$p=\sum_{i=k}^{min(n_{j},n_{i})}\frac{\left(\begin{array}{c} n_{j}\\ k \end{array})(\begin{array}{c} n-n_{j}\\ n_{i}-k \end{array}\right)}{\left(\begin{array}{c} n\\ n_{i} \end{array}\right)}$$ (4)

where

- *n* is a number of target genes in the DiseaseEnhancer database
- *n*_*i*_ is a number of target genes of enhancer *e*_*i*_
- *n*_*j*_ is a number of target genes of enhancer *e*_*j*_
- *k* is a number of shared target genes between two enhancers *e*_*i*_ and *e*_*j*_.

By selecting only enhancer pair having p-value ≤ 0.05, we finally obtained 2,636 significant associations among 539 enhancers. Fig 1(B) illustrates the construction of the enhancer functional interaction network, *G*_*E*_(*V*_*E*_, *E*_*E*_). This network was then represented by an adjacency matrix *W*_*E*_, where an element (*W*_*E*_)_*i*,*j*_ was set to 1 or 0 with respect to whether an interaction between two enhancers *e*_*i*_ and *e*_*j*_ exists or not.

#### 2.1.3 Construction of a heterogeneous network of diseases and enhancers

We collected 1,059 known disease-enhancer associations from the DiseaseEnhancer database [14], which is a comprehensive map of manually curated disease-enhancer associations between 802 enhancers and 167 human diseases. Enhancers in the database are represented by their positions in chromosomes (i.e., start and end positions). Disease names were mapped to DO terms before estimating the similarity between the two diseases. Finally, we constructed 963 DO term-enhancer associations between 122 DO terms and 738 enhancers.

The heterogeneous network of diseases and enhancers was constructed by connecting the enhancer functional interaction network with the disease similarity network by known disease-enhancer associations (Fig 1(C)). Finally, 554 DO term-enhancer associations between 102 diseases and 512 enhancers remained (i.e., diseases and enhancers which do not belong to the disease similarity network and the enhancer functional interaction network respectively were removed). Associations between diseases and enhancers can be considered as a bipartite network. This network was represented by an adjacency matrix *W*_*ED*_, where an element (*W*_*ED*_)_*i*,*j*_ of the matrix represents whether or not an enhancer *e*_*i*_ is known to be associated with a disease *d*_*j*_.

#### 2.1.4 A random walk scheme on networks of diseases and enhancers

In this section, we describe how the random walk with restart algorithm (RWR) used in RWDisEnh can rank candidate enhancers/diseases relatively to a set of enhancers/diseases known to be associated with a disease of interest (*d*)/an enhancer of interest (*e*), respectively.

*A random walk scheme*. Given a connected weighted network *G*(*V*, *E*) with a set of nodes *V* = {*v*_*1*_, *v*_*2*_, *…*, *v*_*N*_}, N is the number of nodes in the network, and a set of links *E* = {(*v*_*i*_, *v*_*j*_)| *v*_*i*_, *v*_*j*_∈*V*}, a set of source nodes *S*⊆*V* and a N×N adjacency matrix *W* of link weights. Here, we introduce algorithms for measuring the relative importance of node *v*_*i*_ to *S*. RWR is a variant of the random walk and it mimics a walker that moves from a current node to a randomly selected adjacent node or goes back to source nodes with a restart-probability *γ*∈(0, 1). RWR equation can be described as follows:

$$P^{t+1}=(1-\gamma )W^{\prime }P^{t}+{\gamma P}^{0}$$ (5)

where *P*^*t*^ is a N×1 probability vector of |*V*| nodes at a time step *t* of which the *i*th element represents the probability of the walker being at node *v*_*i*_∈*V*, and *P*^0^ is the N×1 initial probability vector. *W*′ is the transition matrix of the graph, the (*i*, *j*) element in *W*′, denotes a probability with which a walker at *v*_*i*_ moves to *v*_*j*_ among *V*\{*v*_*i*_}. All nodes in the network are eventually ranked according to the steady-state probability vector *P*^∞^. The steady-state of each node represents its relative importance to the set of source nodes *S*.

The prediction of disease-enhancer associations can be formulated as the ranking of candidate enhancers/diseases by their relative importance measured by the RWR algorithm to a set of source nodes (*S*), where *S* includes enhancers/diseases known to be associated with a disease of interest (*d*)/an enhancer of interest (*e*), respectively. In other words, the relative importance value measures how much a candidate enhancer/disease is associated with *d*/*e* (Fig 1(D)). This algorithm was used for predicting disease-gene associations [19–22,38,39]. In the following sections, we are going to describe more detail on how the RWR algorithm is applied to networks of diseases and enhancers to predict disease-enhancer associations.

*A random walk scheme on the enhancer functional interaction network and the disease similarity network*. The enhancer functional interaction network and the disease similarity network are homogeneous networks, which contain only one type of node (i.e., either enhancer or disease). In the first case, the prediction of disease-enhancer associations is considered in a disease-centric view: Given a disease *d*_*1*_, the goal is to rank all candidate enhancers by their relevance to *d*_*1*_ (Fig 1(D)). Then, the enhancer functional interaction network was used as a homogeneous network of enhancers. Thus, the transition matrix *W*′ is defined as follows:

$${\left(W^{\prime }\right)}_{ij}=\frac{{\left(W_{E}\right)}_{ij}}{\sum_{j}{\left(W_{E}\right)}_{ij}}$$ (6)

where *W*_*E*_ is the adjacency matrix of the enhancer functional interaction network.

In the second case, the prediction of disease-enhancer associations is considered in an enhancer-centric view: Given an enhancer *e*_*3*_, the goal is to rank all candidate diseases by their relevance to *e*_*3*_ (Fig 1(D)). Then, the disease similarity network was used as a homogeneous network of diseases. Thus, the transition matrix *W*′ is defined as follows:

$${\left(W^{\prime }\right)}_{ij}=\frac{{\left(W_{D}\right)}_{ij}}{\sum_{j}{\left(W_{D}\right)}_{ij}}$$ (7)

where *W*_*D*_ is the adjacency matrix of the disease similarity network.

In addition, the set of source nodes (*S*) was specified by enhancers that were known to be associated with *d*_*1*_ in the disease-centric view, and it was specified by diseases known to be associated with *e*_*3*_ in the enhancer-centric view (Fig 1(D)). Then, the initial probability vector was defined as follows:

$$p_{i}^{0}=\left\{ \begin{array}{c} \frac{1}{\left|S\right|}\;if\;v_{i}\in S\\ 0\;otherwise \end{array}\right.$$ (8)

All remaining enhancers/diseases in the homogeneous network of enhancers/diseases were specified as candidate enhancers/diseases, respectively.

*A random walk scheme on the heterogeneous network of diseases and enhancers*. The RWR algorithm can be extended to work on a heterogeneous network of diseases and enhancers. Thus, the transition matrix *W*′ was defined as follows:

$$W^{\prime }=\left[\begin{array}{cc} W_{E}^{\prime } & W_{ED}^{\prime }\\ W_{DE}^{\prime } & W_{D}^{\prime } \end{array}\right]$$ (9)

where $W_{E}^{\prime }$ and $W_{D}^{\prime }$ are intra-subnetwork transition matrices of the enhancer functional interaction network and the disease similarity network, respectively. $W_{ED}^{\prime },\;W_{DE}^{\prime }$ are inter-subnetwork transition matrices. Let *λ* be the jumping probability the random walker jumps from the enhancer functional interaction network to the disease similarity network or vice versa. Then, these matrices were defined as follows:

$${\left(W_{ED}^{\prime }\right)}_{i,j}=p(d_{j}|e_{i})=\left\{ \begin{array}{c} \frac{\lambda {\left(W_{ED}\right)}_{ij}}{\sum_{j}{\left(W_{ED}\right)}_{ij}}\;if\;\sum_{j}{\left(W_{ED}\right)}_{ij}\neq 0\\ 0\;otherwise \end{array}\right.$$ (10)

$${\left(W_{DE}^{\prime }\right)}_{i,j}=p(e_{j}|d_{i})=\left\{ \begin{array}{c} \frac{\lambda {\left(W_{ED}\right)}_{ji}}{\sum_{j}{\left(W_{ED}\right)}_{ji}}\;if\;\sum_{j}{\left(W_{ED}\right)}_{ji}\neq 0\\ 0\;otherwise \end{array}\right.$$ (11)

$${\left(W_{E}^{\prime }\right)}_{i,j}=\left\{ \begin{array}{c} \frac{{\left(W_{E}\right)}_{ij}}{\sum_{j}{\left(W_{E}\right)}_{ij}}\;if\;\sum_{j}{\left(W_{ED}\right)}_{ij}=0\\ \frac{{\left(1-\lambda )(W_{E}\right)}_{ij}}{\sum_{j}{\left(W_{E}\right)}_{ij}}otherwise \end{array}\right.$$ (12)

$${\left(W_{D}^{\prime }\right)}_{i,j}=\left\{ \begin{array}{c} \frac{{\left(W_{D}\right)}_{ij}}{\sum_{j}{\left(W_{D}\right)}_{ij}}\;if\;\sum_{j}{\left(W_{ED}\right)}_{ji}=0\\ \frac{{\left(1-\lambda )(W_{D}\right)}_{ij}}{\sum_{j}{\left(W_{D}\right)}_{ij}}\;otherwise \end{array}\right.$$ (13)

where *W*_*ED*_ is the adjacency matrix of the bipartite network.

In this case, we only consider the prediction of disease-enhancer associations in a disease-centric view: Given a disease of interest *d*_*1*_, the set of source nodes (*S*) was specified by the set of enhancers known to be associated with *d*_*1*_ (*S’*) and *d*_*1*_. By letting *η* be the parameter to weigh the importance of each network, the initial probability vector was defined as follows:

$$p_{i}^{0}=\left\{ \begin{array}{c} (1-\eta )\frac{1}{\left|S\prime \right|}\;if\;v_{i}\in S\prime \\ \eta \;if\;v_{i}\equiv d_{1}\\ 0\;otherwise \end{array}\right.$$ (14)

All remaining enhancers in the enhancer functional interaction network were specified as candidate enhancers.

### 2.2 Baselines

#### 2.2.1 PageRank with Priors

Similar to the RWR algorithm, PageRank with Priors (PRP) [32], an extension of the original Google’s PageRank algorithm [40], is also a network diffusion method. PRP mimics a random Internet surfer starting from one of a set of source nodes (*S*), and follows one of the links randomly in each step. In this process, the surfer jumps back to the source nodes at back-probability *β* ∈(0, 1), thus restarting the whole process. Therefore, this algorithm generates a score that is proportional to the probability of reaching any node on the graph. This score indicates the relative importance of those nodes to the source nodes. Given the enhancer functional interaction network, *G*_*E*_(*V*_*E*_, *E*_*E*_), and a set of known enhancers (*S*) associated with a disease of interest *d*, each candidate enhancer was assigned a score representing its relative importance to *S*. Then, the candidate enhancers were ranked by their score. Formally, the PRP algorithm can be described as follows:

$$p_{i}^{t+1}=(1-\beta )\left(\sum_{j\in {\left({V_{E}}_{in}\right)}_{i}}{p_{ji}p}_{j}^{t}\right)+\beta p_{i}^{0}$$ (15)

where (${V_{E}}_{in}$)_*i*_ is a set of incoming enhancers of *e*_*i*_, *p*_*ji*_ is the probability of the random surfer arriving *e*_*i*_ from *e*_*j*_. *p*_*ji*_ is defined as follow:

$$p_{ji}=\frac{{\left(W_{E}\right)}_{ji}}{\sum_{k\in {\left({V_{E}}_{out}\right)}_{j}}{\left(W_{E}\right)}_{jk}}$$ (16)

where (${V_{E}}_{out}$)_*j*_ is a set of outgoing enhancers of *e*_*j*_.

Similar to the random walk scheme on the enhancer functional interaction network, *p*_*i*_^*0*^ is the initial probability of *e*_*i*_ and is assigned to zero or 1/|*S*| if *e*_*i*_ is a non-source node or a source node, respectively (Eq 8). In addition, all remaining enhancers in the enhancer functional interaction network were specified as candidate enhancers.

For running on the heterogeneous network of diseases and enhancers, *W*_*E*_ in Eq 16 was replaced by *W’* in Eq 9. Given a disease of interest *d*_*1*_, the set of source nodes (*S*) was specified by the set of enhancers known to be associated with *d*_*1*_ (*S’*) and *d*_*1*_, and then *p*_*i*_^*0*^ is set as in Eq 14.

#### 2.2.2 MaxLink

In addition to the network diffusion methods, i.e., RWR and PRP, we investigated a neighborhood-based method, MaxLink [33,34]. Given a disease of interest (*d*_*1*_), the neighborhood-based algorithm was based on direct neighbors of source nodes (*S*) (i.e., known *d*_*1*_-associated enhancers in the enhancer functional interaction network, *G*_*E*_(*V*_*E*_, *E*_*E*_)). MaxLink considers neighbors of *S* as candidate enhancers and assigns to each candidate (*v*_*i*_) a score corresponding to the number of links to *S* (*ML*). This score is used for ranking the candidate enhancers. To avoid highly connected nodes from receiving high ranking, which are solely based on their high degree (deg(*v*_*i*_)), MaxLink discards candidates with connection probability ≥ 0.5, where the connection probability was defined as follows:

$$connection\;probability\;(v_{i})=\frac{\left(\begin{array}{c} \left|S\right|\\ ML \end{array})(\begin{array}{c} \left|V_{E}|-|S\right|\\ deg\;(v_{i})-ML \end{array}\right)}{\left(\begin{array}{c} \left|V_{E}\right|\\ deg\;(v_{i}) \end{array}\right)}$$ (17)

For running on the heterogeneous network of diseases and enhancers, *G*_*E*_(*V*_*E*_, *E*_*E*_) was replaced by *G*(*V*, *E*). Similarly, given a disease of interest *d*_*1*_, the set of source nodes (*S*) was specified by the set of enhancers known to be associated with *d*_*1*_ (*S’*) and *d*_*1*_, then neighbors of *S’* were set as candidate enhancers.

### 2.3 Performance evaluation

To assess the prediction performance of ranking methods (i.e., RWDisEnh and baselines) on different networks of diseases and enhancers, we used the leave-one-out cross-validation (LOOCV) method for each disease/enhancer depending on disease/enhancer-centric view. More specifically, for the disease-centric view with each disease (*d*) with known associated enhancers (*S*), in each round of LOOCV, we held out one known *d*-associated enhancer. The held-out enhancer (*s*) and remaining enhancers (*C*) in the enhancer functional interaction network, which were not known to be associated with *d*, were then ranked by the method. After that, we plotted the receiver operating characteristic (ROC) curve and calculated the area under the curve (AUC) to compare the performance of the methods. This curve represents the relationship between *sensitivity* and (1-*specificity*), where *sensitivity* refers to the percentage of known *d*-associated enhancers that were ranked above a particular threshold, and *specificity* refers to the percentage of enhancers that were not known to be associated top-ranked below this threshold. More specifically, given a threshold *τ*, we counted TP (true positives), FN (false negatives), FP (false positives), and TN (true negatives), which were formally defined as follows:

$$P=\sum_{s\in S}I(rank(s)\leq \tau )\;FN=\sum_{s\in S}I(rank(s)>\tau )$$ (18)

$$FP=\sum_{c\in C}I(rank(c)\leq \tau )\;TN=\sum_{c\in C}I(rank(c)>\tau )$$ (19)

where *rank*(*s*), *rank*(*c*), and *I*(∙) denote the rank of *s*, the rank of an enhancer *c* out of the set *C*, and the indicator function, respectively. Then, we defined *sensitivity* and (1-*specificity*) as follows:

$$sensitivity=\frac{TP}{TP+FN}$$ (20)

$$1-specificity=\frac{FP}{FP+TN}$$ (21)

By varying *τ* from one to the number of enhancers in the set *C*∪{*s*}, the relationship between *sensitivity* and (1-*specificity*) was plotted. The ROC curve is the curve constructed based on those pairs of values, and the AUC is the area under the ROC curve. For the enhancer-centric view, we repeat the same procedure for each enhancer.

## 3. Results

### 3.1 Parameter settings

**Fig 2:**
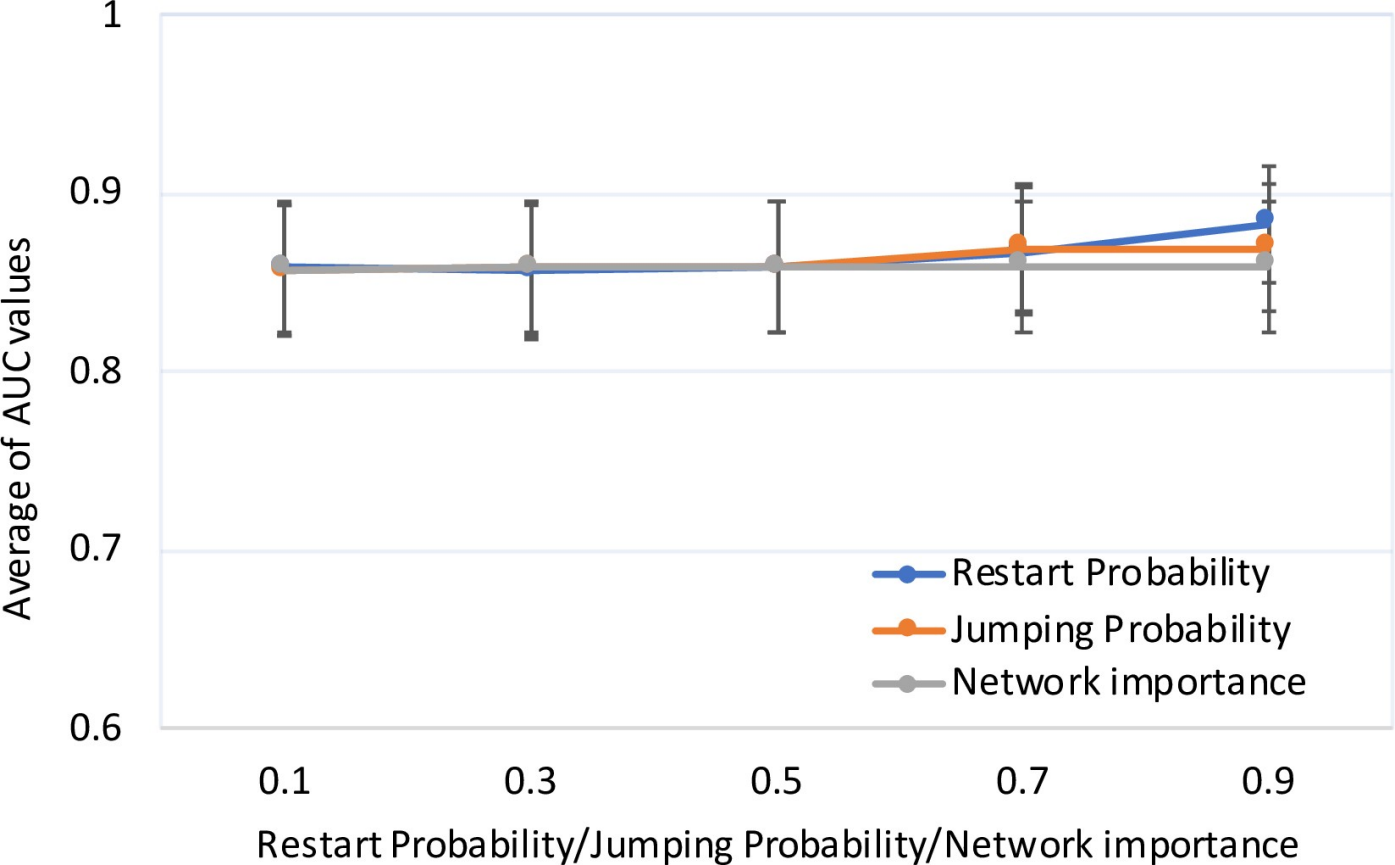
Prediction performance of RWDisEnh on the heterogeneous network of diseases and enhancers with different parameter settings. Restart Probability: *λ = η* = 0.5, and *γ* in {0.1, 0.3, 0.5, 0.7, 0.9}. Network importance: *λ = γ* = 0.5, and *η* in {0.1, 0.3, 0.5, 0.7, 0.9}; and Jumping Probability: *η = γ* = 0.5, and *λ* in {0.1, 0.3, 0.5, 0.7, 0.9}. Average AUC values and standard errors were calculated based on the set of diseases for each data point.

To estimate the prediction performance of RWDisEnh on the heterogeneous network of diseases and enhancers, we varied parameters *λ*, *η*, and *γ* in a range of (0, 1). First, we kept *λ = η* = 0.5, and varied *γ* in {0.1, 0.3, 0.5, 0.7, 0.9}. Second, we kept *λ = γ* = 0.5, and varied *η* in {0.1, 0.3, 0.5, 0.7, 0.9}. Third, we kept *η = γ* = 0.5, and varied *λ* in {0.1, 0.3, 0.5, 0.7, 0.9}. Then, we used the LOOCV scheme for each disease in the set of 102 diseases, which have at least one known disease-associated enhancer in the enhancer functional interaction network. Finally, the performance of RWDisEnh was summarized as the average of AUC values over the entire set of diseases. Fig 2 shows that the prediction performance of RWDisEnh was mostly stable against the change of parameters. The minimal performance was 0.856 and achieved at *λ = η* = 0.5 and *γ* = 0.3, meanwhile the maximal one was 0.883 when *λ = η* = 0.5 and *γ* = 0.9. Fig 2 also shows that when *γ* increased, the prediction performance was increased. This indicates that disease-associated enhancers tend to closely interact with each other. When *η* was varied, the prediction performance was changed slightly in a range of (0.857, 0.859), indicating that RWDisEnh was stable with the change of *η*. For the change of *λ* when *γ = η* = 0.5, RWDisEnh performed slightly better when *λ* increased. More specifically, RWDisEnh achieved worst (AUC = 0.857) and best (AUC = 0.869) performance at *λ* = 0.1 and *λ* = 0.7, respectively. This meant that if we force the random walker tends to jump from the enhancer functional interaction network to the disease similarity network, then RWDisEnh archived better performance.

### 3.2 RWDisEnh on networks of diseases and enhancers

In this section, we demonstrate the effects of the enhancer functional interaction network and the disease similarity network (i.e., the homogeneous networks) on the prediction performance of RWDisEnh. To this end, we compared the prediction performance of RWDisEnh on the heterogeneous network of diseases and enhancers (shortly called the heterogeneous network) with that on the homogeneous networks. The prediction performance of RWDisEnh was also assessed using the LOOCV scheme.

**Fig 3:**
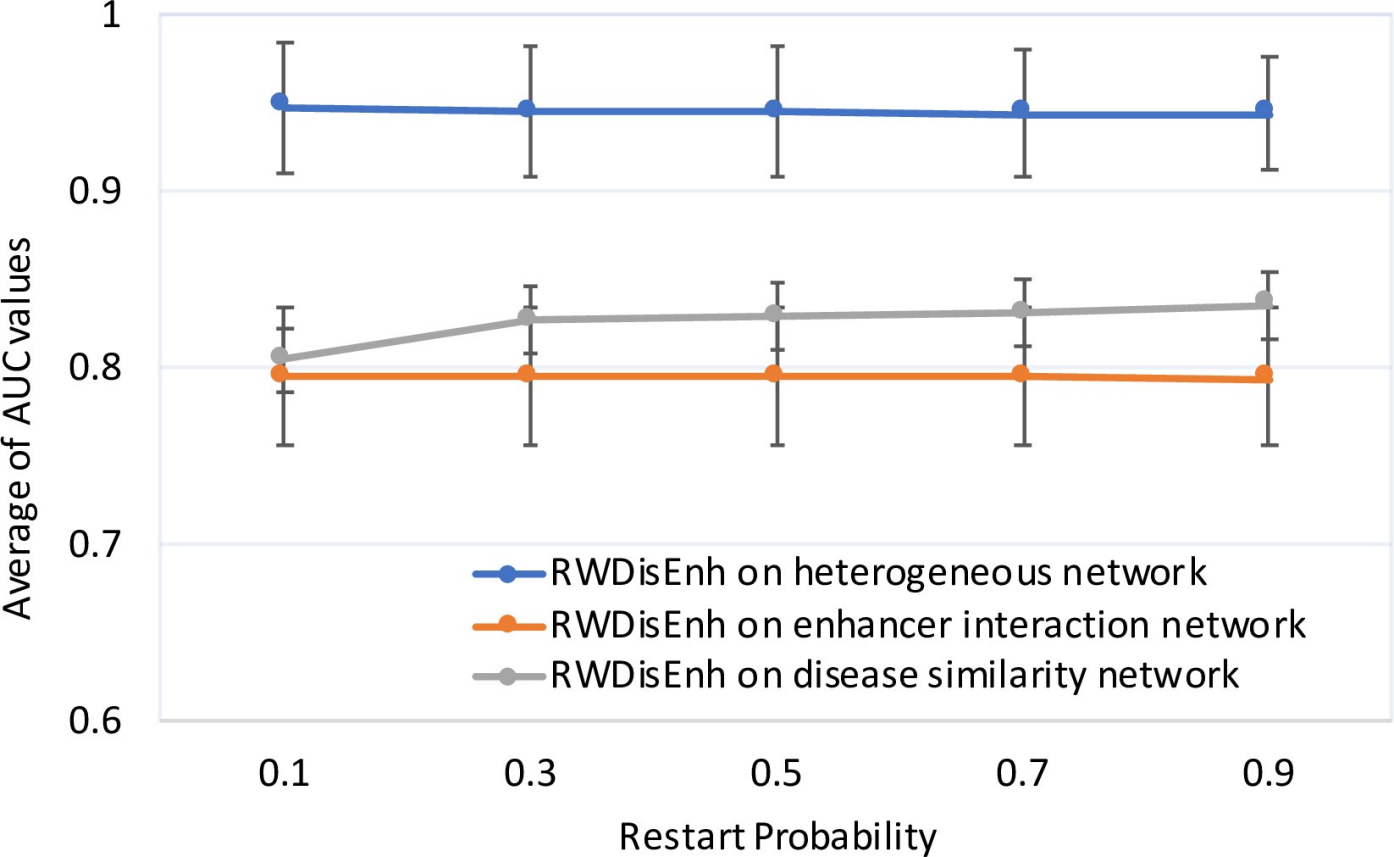
Performance comparison of RWDisEnh on the heterogeneous network of diseases and enhancers and that on the enhancer functional interaction network and the disease similarity network. *λ* and *η* are set to 0.5 for RWDisEnh running on the heterogeneous network of diseases and enhancers, *γ* is varied in {0.1, 0.3, 0.5, 0.7, 0.9} for the three networks. Average AUC values and standard errors were calculated based on the set of diseases/enhancers for each data point.

First, we assessed the prediction performance of RWDisEnh on the heterogeneous network and the enhancer functional interaction network. Due to the stability of RWDisEnh on the heterogeneous network, we set *λ* = *η* = 0.5, and varied *γ* in {0.1, 0.3, 0.5, 0.7, 0.9} for the comparison. By using the LOOCV scheme, only diseases having at least two known associated enhancers were satisfied for experiments with RWDisEnh on the enhancer functional interaction network. Thus, 54 of 102 diseases were used for the analysis of the two networks for a fair comparison. Fig 3 shows that the prediction performance of RWDisEnh on the two networks is stable when *γ* is changed. More importantly, the prediction performance of RWDisEnh on the heterogeneous network was better than that on the enhancer functional interaction network (*i*.*e*., average AUC values were 0.945 and 0.795 for the heterogeneous and the enhancer functional interaction networks, respectively; p-value = 1.00 × 10^−9^ using *t*-Test: Two-sample assuming unequal variances). This indicated that without the disease similarity network, RWDisEnh performed relatively poorer compared to the case the disease similarity network was integrated with the enhancer functional interaction network in the heterogeneous network. This also demonstrated the important role of the disease similarity network in predicting novel disease-enhancer associations.

Second, we assessed the prediction performance of RWDisEnh on the disease similarity network. Similarly, only 47 enhancers having at least two known associated diseases were valid for LOOCV analysis on the disease similarity network. More specifically, for each enhancer *e*, in each round of LOOCV, we held out one disease known to be associated with *e*. The rest of the known diseases associated with enhancer *e* were used as seed nodes (*S*). The held-out disease and remaining diseases in the disease similarity network, which were not known to be associated with e, were ranked by RWDisEnh. Then, the ROC curve was constructed, and the AUC was used to assess the performance of RWDisEnh on the disease similarity network. Similarly, the performance of RWDisEnh was summarized as the average of AUC values over the entire set of 47 enhancers. Fig 3 also shows that the prediction performance of RWDisEnh is higher when *γ* is increased. More importantly, RWDisEnh performs better on the heterogeneous network than on the disease similarity network (*i*.*e*., average AUC values were 0.945 and 0.826 for the heterogeneous and the disease similarity networks, respectively; p-value = 1.24 × 10^−5^ using *t*-Test: Two-sample assuming unequal variances). This means that when the enhancer functional interaction network was absent, then the prediction performance of RWDisEnh was decreased significantly. This also indicates that enhancer interactions also significantly contributed to the prediction performance. Taken together, disease similarity and enhancer functional interaction information helped improve the prediction performance of disease-enhancer associations.

Moreover, RWDisEnh performed better on the disease similarity network than on the enhancer functional interaction network (*i*.*e*., average AUC values were 0.826 and 0.795 for the disease similarity network and the enhancer functional interaction network, respectively; p-value = 2.38 × 10^−3^ using *t*-Test: Two-sample assuming unequal variances). Together with the previous observation that the prediction performance of RWDisEnh was increased when the random walker tends towards the disease similarity network, this result indicates that the disease similarity network contributed more to the prediction performance than the enhancer functional interaction network.

### 3.3 Performance comparison between RWDisEnh and other methods

To our knowledge, HEDD [15] is the first computational method proposed for predicting disease-enhancer associations. The method in HEDD was based on an assumption that if an enhancer targets a known disease-associated gene, then this enhancer is functionally connected to the disease. Therefore, HEDD relied on known enhancer-target gene and known disease-gene associations. Formally, let *P*_*EG*_ be the probability of association between an enhancer and a gene, and *P*_*GD*_ be the probability of association between a gene and a disease. Then, the probability of association between the enhancer and the disease is *P*_*ED*_ = *P*_*EG*_ × *P*_*GD*._ Since HEDD did not rely on known disease-enhancer associations when scoring a pair of enhancer and disease; thus, it was not suitable to be selected for the comparison with our method based on the LOOCV scheme.

**Fig 4:**
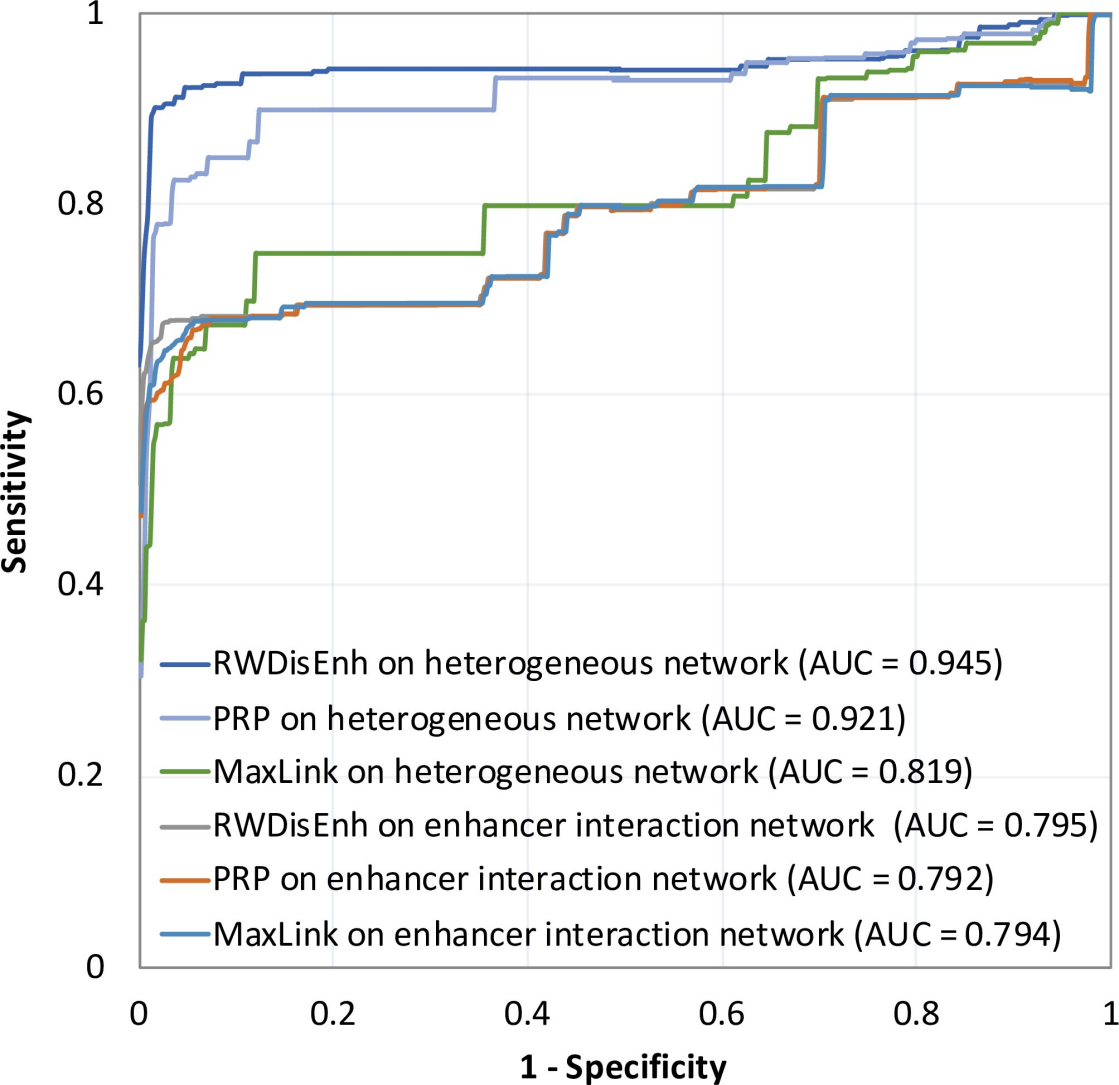
Performance comparison between RWDisEnh and other methods. The best settings of RWDisEnh and PRP were used.

Therefore, we compared RWDisEnh with another network diffusion method, i.e., PageRank with Priors (PRP) [32], and a neighborhood-based method, i.e., MaxLink [33,34] based on their best settings. Due to stability of the performance, *γ* is set to 0.5 for RWDisEnh on both the heterogeneous network and the functional enhancer interaction; meanwhile, *γ* is set to 0.9 for RWDisEnh on the disease similarity network. Meanwhile, PRP archived the best performance with the back-probability *β* = 0.7. Fig 4 shows the prediction performance of the three methods in terms of ROC and AUC values on the heterogeneous network and the enhancer functional interaction network. The results on the heterogeneous network indicate that RWDisEnh (AUC = 0.945) is better than the PRP (AUC = 0.921) and the MaxLink method (AUC = 0.819). Interestingly, the prediction performance of RWDisEnh (AUC = 0.795) and PRP (AUC = 0.792) on the enhancer functional interaction network are comparable with that of the MaxLink method (AUC = 0.794). This supports our assumption that neighbors of known disease-associated enhancers (i.e., they share target genes) can be promising disease enhancers. Thus, although the neighborhood-based method locally searches neighbors of known disease-associated enhancers for novel disease enhancers, it achieves comparable performance with the global methods (i.e., RWDisEnh and PRP) on the same network.

### 3.4 Prediction of novel disease-associated enhancers

Besides showing the overall performance of RWDisEnh on predicting disease-enhancer associations based on known disease-enhancer associations using the LOOCV scheme, we here demonstrate its ability in predicting novel disease-associated enhancers. In particular, for each in the set of 102 diseases, we used RWDisEnh on the heterogeneous network to rank all candidate enhancers (*i*.*e*., enhancers which were not known to be associated with a disease of interest). Then, we selected the top 10 ranked candidate enhancers of each disease for the evidence search.

#### 3.4.1 Search for direct evidence

We collected evidence of the association between single nucleotide polymorphisms (SNPs) located in the top-ranked candidate enhancers and diseases from genome-wide association studies. To this end, we used PhenoScanner [41,42], a curated database of publicly available results from large-scale genetic association studies in humans. PhenoScanner helps scan more than 65 billion genotype-phenotype associations for over 150 million genetic variants. By using a genomic region search, we found 12 direct associations between four diseases and 12 enhancers (Table 1). For example, a genome-wide association study in the Japanese population [43] (PubMed ID: 26818947) identified rs1421085, located in enhancer chr16:53799602–53801200, significantly (p-value = 4.0 x 10^−15^) associated with type 2 diabetes. A SNP rs10877013 (p-value = 7.2 x 10^−6^) in enhancer chr12:58163402–58165600 was found to be associated with rheumatoid arthritis [44] (PubMed ID: 24390342). A comprehensive genome-wide association meta-analysis found several SNPs located in eight enhancers (i.e., chr9:22071264–22073264, chr9:22072402–22073600, chr9:22075795–22077795, chr9:22093330–22095330, chr9:22096002–22099600, chr9:22101602–22110600, chr9:22110602–22120000 and chr9:22123402–22125200) associated with myocardial infarction [45] (PubMed ID: 26343387). Finally, SNPs in two enhancers (i.e., chr17:59234602–59239400 and chr9:21974127–21976127) were found to be associated with coronary heart disease [46] (PubMed ID: 28714975) [47] (PubMed ID: 29212778).

**Table 1:** Enhancers directly associated with four diseases from genome-wide association studies.

Disease	Enhancer	Target Genes	Location	SNP ID (P-value)	PubMed ID
type-2 diabetes	chr16:53799602–53801200	IRX5, IRX3	Intron	rs1421085 (4.0E-15)	26818947
rheumatoid arthritis	chr12:58163402–58165600	TSPAN31, CYP27B1, TSFM, AVIL, FAM119B	Intron	rs10877013 (7.2E-6)	24390342
myocardial infarction	chr9:22071264–22073264	CDKN2A	Intron	rs10757269 (1.483E-55), rs10757270 (8.832E-53), rs4451405 (1.476E-8), rs4645630 (2.271E-9), rs9632884 (4.005E-56), rs9632885 (9.062E-25)	26343387
	chr9:22072402–22073600	CDKN2A	Intron	rs10757270 (8.832E-53), rs9632885 (9.062E-25)	26343387
	chr9:22075795–22077795	CDKN2A	Intron	rs10757271 (2.531E-57), rs10811652 (1.646E-58), rs1412832 (3.349E-8), rs1831733 (1.702E-65)	26343387
	chr9:22093330–22095330	CDKN2A	Intron	rs10738608 (1.424E-70), rs4977757 (1.69E-67)	26343387
	chr9:22096002–22099600	CDKN2A	Intron	rs4977574 (1.1E-18)	17478679
				rs4977574 (3.0E-44)	19198609
				rs2891168 (1.32E-7), rs4977574 (1.32E-7)	21088011
				rs4977574 (1.02E-19)	21378990
				rs4977574 (8.0E-6)	24916648
				rs10757274 (1.331E-73), rs1537371 (9.721E-71), rs2891168 (5.0E-75), rs2891168 (5.636E-75), rs4977574 (4.584E-75)	26343387
	chr9:22101602–22110600	CDKN2A	Intron	rs6475608 (6.3E-8)	17478679
				rs1333042 (1.32E-7)	21088011
				rs10757275 (3.753E-74), rs1333042 (6.798E-71), rs1333043 (3.64E-70), rs1412834 (8.419E-15), rs1537372 (1.089E-64), rs1537373 (2.883E-71), rs62555370 (2.64E-15), rs62555371 (2.846E-15), rs6475609 (9.021E-15), rs7859362 (2.365E-70), rs7859727 (1.119E-70)	26343387
	chr9:22110602–22120000	CDKN2A, ANRIL	Intron	rs1333045 (6.3E-15), rs2383207 (1.0E-16)	17478679
				rs944797 (1.2E-14)	21971053
				rs1004638 (5.919E-15), rs10511701 (5.069E-68), rs10733376 (1.097E-14), rs10738609 (1.52E-72), rs1333045 (3.282E-14), rs1537374 (5.113E-15), rs1537375 (5.988E-68), rs1537376 (8.325E-16), rs2383206 (8.557E-16), rs2383207 (5.512E-15), rs7341786 (1.214E-14), rs7341791 (1.226E-14), rs944797 (1.334E-15)	26343387
	chr9:22123402–22125200	CDKN2A, MTAP, CDKN2B-AS1, CDKN2B	downstream	rs10757278 (1.0E-20), rs1333046 (2.5E-17)	17478679
				rs10738610 (3.611E-73), rs10757277 (9.542E-71), rs10757278 (1.041E-70), rs10757279 (1.209E-70), rs10811656 (3.856E-65), rs1333046 (1.734E-73), rs1333047 (1.078E-66), rs4977575 (9.325E-67), rs7857118 (3.09E-69)	26343387
coronary heart disease	chr17:59234602–59239400	TBX4, BCAS3, TBX2, NACA2	Intron	rs1476098 (6.27E-6), rs1476099 (7.31E-6), rs2041302 (7.43E-6), rs2159373 (5.88E-6), rs2378816 (9.24E-6), rs9905761 (6.72E-6)	28714975
				rs11868441 (3.593E-9), rs11868441 (3.8E-6), rs1476098 (1.2E-6), rs1476098 (8.514E-10), rs1476098 (9.0E-10), rs1476099 (1.3E-6), rs1476099 (9.489E-10), rs2041302 (1.152E-9), rs2041302 (2.0E-6), rs2159373 (1.103E-9), rs2159373 (1.9E-6), rs2378816 (3.383E-9), rs2378816 (3.8E-6), rs8075455 (2.063E-9), rs8075455 (2.4E-6), rs9905761 (1.405E-9), rs9905761 (2.2E-6)	29212778
	chr9:21974127–21976127	CDKN2A	Intron	rs3731239 (4.224E-7)	21378988
				rs36228834 (1.67E-8), rs3731239 (7.55E-14)	26343387
				rs36228834 (4.65E-9), rs3731239 (1.25E-17)	28714975
				rs36228503 (2.624E-6), rs36228834 (1.833E-15), rs36228834 (6.8E-9), rs3731238 (2.997E-6), rs3731239 (1.8E-36), rs3731239 (1.916E-47)	29212778

#### 3.4.2 Search for indirect evidence

In addition to direct evidence from genome-wide association studies, we here search for indirect evidence from the literature for the top-ranked candidate enhancers. Genetic variants of enhancers contribute important roles in disease progression because enhancers are regulatory elements that alter the expression level of their target genes. Aberrant activity of enhancers may result in diseases, *i*.*e*., cancers [1,10]. Therefore, to support potential associations between top-ranked enhancers with a disease, we collected evidence from the literature that indicates changes in gene expression of target genes of the enhancers associated with the disease of interest. Finally, we found evidence of the association with 22 diseases for 37 enhancers (S1 Table). For instance, it was shown that a higher expression level of ID2 (i.e., the target gene of enhancer chr2:8440002–8455200) was associated with advanced breast cancer [48]. CCL2 (targeted by enhancer chr17:32559802–32586800) is important for regulating cell growth and survival by inhibiting necrosis and autophagy and is overexpressed in luminal B breast cancer cells and [49]. For endometrial cancer, it was indicated that high expression of PTEN targeted by enhancer chr17:5519002–5520600 is positively correlated with myometrial invasion in endometrial cancer [50]. Expression of BCL2 (targeted by two enhancers chr15:54202002–54203000 and chr15:54203002–54203600) is significantly more frequent in early clinical stages in both types of endometrial cancer [51]. A high expression level of SOX9 is associated with gastric cancers [52]. Guo et al., 2012 showed that the overexpression of SOX9 protein in hepatocellular carcinoma tissues is of predictive value on tumor progression and poor prognosis [53]. For lung cancer, BCL11A (targeted by chr2:60719002–60776000) overexpression predicts survival and relapse in non-small cell lung cancer [54]. Finally, up-regulated p16 expression may represent a unique feature of aggressive neuroblastoma [55].

## 4. Conclusions and discussion

Enhancers regulate their target genes; thus aberration on enhancers could change the expression level of the target genes, and consequently may cause diseases. To date, millions of enhancers have been discovered, yet our understanding of their associations to diseases is very limited. DiseaseEnhancer [14] is a pioneer in collecting disease-enhancer associations by literature curation. Considering the huge amount of enhancers and diversity of diseases, computational methods are needed to narrow down a list of potential disease-associated enhancers. The computational methods score the degree of association between diseases and enhancers. Based on these scores, enhancers/diseases are ranked, then top-ranked enhancers/diseases can be selected as promising candidates for further analyses. In this study, we assumed that functional interacting enhancers are associated with similar diseases. Therefore, we developed a computational method to exploit the similarities among diseases and functional interactions among enhancers. More specifically, the degree of association between a disease and an enhancer was globally measured by a random walk scheme on networks of diseases and enhancers. The experimental results showed that our method achieved the best performance in terms of AUC value when the disease similarity network and the enhancer functional interaction network were used simultaneously. Also, our method performed better than the other network diffusion and the neighborhood-based method, which locally searches neighbors of known disease-associated enhancers for novel ones. Finally, we applied our method to find potential enhancers associated with 102 diseases. A total of 12 enhancers was found directly to be associated with four diseases from genome-wide association studies. Besides, we found indirect evidence from the literature for 37 enhancers that changes in the expression of their target genes are associated with 22 diseases.

Finally, network-based methods have been shown to be dominant ones for various biomedical problems. For example, HotNet was proposed for predicting significantly mutated pathways and subnetworks associated with clinical data in cancer [56,57]; and graph kernel diffusion methods [58], e.g., Gaussian Kernel [59,60] and Laplacian Exponential Diffusion Kernel [61], were successfully used for the prediction of disease-gene associations. In addition, other approaches for predicting associations have been also applied for predicting non-coding RNA-disease association [62–65] and synergistic drug combinations [66]. Therefore, the adoption of those methods for the prediction of disease-enhancer associations could be a potential direction in future studies. In addition, by constructing more comprehensive networks of diseases, enhancers, and their target genes such as a tripartite network of them, multiplex networks of enhancers and diseases, or a combination between a multiplex and a heterogeneous network, the RWR algorithm could help predict disease-enhancer associations more effectively [17].

## Supporting information

S1 Table Enhancers indirectly associated with 22 diseases with evidence from the literature search. (PDF)
