# Supplementary material for: A network-based method for predicting disease-associated enhancers
Source: PLoS One. 2021 Dec 8;16(12):e0260432. doi: 10.1371/journal.pone.0260432 (PMC8654176; doi:10.1371/journal.pone.0260432)
Supplement: S1 Table — (PDF) [file pone.0260432.s001.pdf]

**Table S1: Enhancers indirectly associated with 22 diseases with evidence from the literature search**

| No. | Disease | Enhancers | Gene Symbol (Entrez ID) | PubMed ID | Information |
|---|---|---|---|---|---|
| 1 | *autism spectrum disorder* | | | | |
| | | chr13:47471402-47471600 | HTR2A (3356) | 24968012 | Data suggest that the HTR2A functional rs6311 polymorphism, which other studies have associated with differential HTR2A mRNA expression, may modulate the severity of depression symptoms in children with autism spectrum disorder. |
| 2 | *bipolar disorder* | | | | |
| | | chr3:119814602-119822600 | GSK3B (2932) | 25041379 | GSK3B protein and mRNA expression were decreased in the dorsolateral prefrontal cortex and temporal lobe of bipolar disorder patients compared to schizophrenia patients and controls. |
| 3 | *breast cancer* | | | | |
| | | chr9:89720602-89726000 | DAPK1 (1612) | 21965790 | DAPK1 expression did not show any meaningful value in predicting outcome for patients with breast cancer. |
| | | | | 26075823 | high DAPK1 expression causes increased cancer cell growth and enhanced signaling via the mTOR/S6K pathway; evaluation of breast cancer patient data sets revealed that high DAPK1 expression associates with worse outcomes in women with p53-mutant cancers |
| | | | | 28231808 | Results show that DNA demethylation of distinct promoter regions is associated with re-expression of the tumor suppressor gene DAPK1. Its knockdown promotes tumor cell migration in breast cancer cell line. |
| | | | | 28429233 | A significant correlation between changes in the levels of expression and methylation was detected for the three apoptosis-regulatory genes (APAF1, DAPK1, and BCL2). The results suggest that methylation play an important role in the regulation of the apoptosis system genes in breast cancer. |
| | | chr2:8440002-8455200 | ID2 (3398) | 23645773 | Higher ID2 expression was associated with advanced breast cancer. |
| | | chr2:60719002-60776000 | BCL11A (53335) | 27774950 | The BCL11A protein is highly expressed in breast cancer and knock-down of BCL11A promotes the apoptosis of MDA-MB-231 cells. |
| | | chr17:32559802-32586800 | CCL2 (6347) | 18790652 | observations support a major tumor-promoting role for co-expression of the chemokines in breast malignancy, and agree with the significant association of joint RANTES and MCP-1 expression with advanced stages of breast cancer. |
| | | | | 25744294 | CCL2 expression is overexpressed in luminal B breast cancer cells and is important for regulating cell growth and survival by inhibiting necrosis and autophagy. |
| | | | | 28077158 | Constitutive expression of CCL2 by the mouse mammary epithelium induces a state of low level chronic inflammation that increases stromal density and elevates cancer risk. We propose that CCL2-driven inflammation contributes to the increased risk of breast cancer observed in women with high mammographic density. |
| | | | | 29107385 | these findings collectively indicate that TGF-beta regulates CCL2 expression in a stage-dependent manner during breast cancer progression |
| 4 | *chronic obstructive pulmonary disease* | | | | |
| | | chr5:110175802-110176800 | TSLP (85480) | 18684970 | There is distinct airways expression of TSLP and chemokines which preferentially attract T helper (Th) type 1- and Th2-type T cells, and influx of T cells bearing their receptors in asthma and chronic obstructive pulmonary disease. |
| 5 | *cleft palate* | | | | |
| | | chr9:100551602-100552200<br>chr9:100554802-100556800<br>chr9:100556802-100557800 | FOXE1 (2304) | 19192046 | A novel homozygous polymorphism that prevented the binding of MYF-5 to FOXE1 promoter and affected the FOXE1 expression was found in 45% nonsyndromic cleft palate. |
| 6 | *endometrial cancer* | | | | |
| | | chr17:5519002-5520600 | PTEN (5728) | 15453811 | PTEN mRNA and protein expression as well as PTEN-related cell growth inhibition in endometrial cancer cells. |
| | | | | 17490733 | In Ishikawa H cells that model type I endometrial cancer in the loss of PTEN and RB1, re-expressing PTEN and RB1 increased the apoptotic and G1 phases and decreased the S and G2-M phases, which further sensitize the cells to gefitinib. |
| | | | | 17924977 | High levels of p-AKT expression occurred independently of the presence of PTEN or PIK3CA mutations in endometrial cancer. |
| | | | | 24929707 | The study demonstrates that the expression of PTEN is directly regulated by miR-205 in endometrial cancer cells and leads to the inhibition of cellular apoptosis. |
| | | | | 26511107 | GAS5 acts as an tumor suppressor lncRNA in endometrial cancer. Through inhibiting the expression of miR-103, GAS5 significantly enhanced the expression of PTEN to promote cancer cell apoptosis. |
| | | | | 26802879 | Study shows that women with polycystic ovarian syndrome and endometrial cancer have an increased endometrial expression of genes (IGF1, IGFBP1 and PTEN) involved in the insulin signaling pathway compared with control women. |
| | | | | 26894937 | High expression of PTEN was positively correlated with myometrial invasion in endometrial cancer. |
| | | chr15:54202002-54203000<br>chr15:54203002-54203600 | BCL2 (596) | 22252757 | BCL-2 expression was significantly more frequent in early clinical stages in both types of endometrial cancer |
| | | chr17:68734530-68736530<br>chr17:68746322-68748322 | SOX9 (6662) | 27262401 | These findings indicate that chronic overexpression of Sox9 in the uterine epithelium can induce the development of endometrial hyperplastic lesions. Thus, SOX9 expression may be a factor in the formation of endometrial cancer. |
| 7 | *endometriosis* | | | | |
| | | chr1:241012202-241013200<br>chr9:22071264-22073264<br>chr9:22072402-22073600<br>chr9:22075795-22077795<br>chr9:22093330-22095330<br>chr9:22096002-22099600<br>chr9:22101602-22110600 | CDKN2A (1029) | 16616093 | Differences in oncoprotein expression between endometriotic and adenomyotic tissues provide further evidence that the pathogenesis of endometriosis is different from that of adenomyosis. |
| 8 | *gastric cancer* | | | | |
| | | chr20:7105289-7107289 | BMP2 (650) | 25698539 | C23 protein meditates bone morphogenetic protein-2-mediated epithelial-to-mesenchymal transition via up-regulation of Erk1/Erk2 and Akt in gastric cancer.Correlations between C23, BMPRII expression and prognosis of gastric cancer patients. |
| | | chr17:68734530-68736530<br>chr17:68746322-68748322 | SOX9 (6662) | 23812904 | High expression of SOX9 is associated with gastric cancers. |
| 9 | *hepatocellular carcinoma* | | | | |
| | | chr17:68697977-68699977 | SOX9 (6662) | 22515642 | Our data suggest for the first time that the overexpression of SOX9 protein in hepatocellular carcinoma tissues is of predictive value on tumor progression and poor prognosis |
| 10 | *leprosy* | | | | |

| | | | | | |
|---|---|---|---|---|---|
| | | chr6:31534802-31548600 | TNF (7124) | 26829382 | Results find that TNF mRNA expression is higher in leprosy patients compared to endemic controls, but does not differ significantly between clinical subgroups. Across leprosy patients, carriage of the minor A allele is associated with low TNF mRNA. Nevertheless, no evidence was found for either allele at this SNP as a risk factor for leprosy per se, or for any subgroups. |
| ***11*** | ***liver cancer*** | | | | |
| | | chr1:59249452-59251452<br>chr1:59250097-59252097 | JUN (3725) | 27341307 | The positive feedback regulation of OCT4 and c-JUN, resulting in the continuous expression of oncogenes such as c-JUN, seems to play a critical role in the determination of the cell fate decision from induced pluripotent stem cells to cancer stem cells in liver cancer. |
| ***12*** | ***lung cancer*** | | | | |
| | | chr2:60719002-60776000 | BCL11A (53335) | 23758992 | BCL11A overexpression predicts survival and relapse in non-small cell lung cancer and is modulated by microRNA-30a and gene amplification. |
| ***13*** | ***neuroblastoma*** | | | | |
| | | chr9:21974127-21976127 | CDKN2A (1029) | 11705866 | Up-regulated p16 expression may represent a unique feature of aggressive neuroblastoma. |
| | | chr11:2190002-2193400 | TH (7054) | 12358785 | Gene expression found upregulated by Glial cell line-derived neurotrophic factor in human neuroblastoma cell lines |
| | | | | 12576454 | Gene expression is a sensitive and semiquantitative marker for minimal residual disease detection of neuroblastoma |
| | | | | 18814238 | High expression of TH both in peripheral blood and bone marrow corresponds to metastatic neuroblastoma at diagnosis, residual disease, and poor outcome. |
| | | | | 19125082 | High tyrosine hydroxylase expression is associated with Minimal residual disease in peripheral blood stem cell harvests from high-risk neuroblastoma. |
| | | | | 19285077 | SIRT1 regulates tyrosine hydroxylase expression and differentiation of neuroblastoma cells via FOXO3a. |
| | | | | 27034145 | The purpose of this study is to investigate the clinical significance of tyrosine hydroxylase (TH) expression in peripheral blood (PB) at diagnosis in patients with neuroblastoma.. The treatment intensity should be tailored according to TH expression in PB at diagnosis. |
| ***14*** | ***obesity*** | | | | |
| | | chr3:123036602-123074000 | ADCY5 (111) | 25793868 | changes in adipose tissue ADCY5 expression are related to obesity and fat distribution. |
| ***15*** | ***ovarian cancer*** | | | | |
| | | chr14:105232802-105261000 | AKT1 (207) | 18231751 | PTEN restores drug sensitivity to cisplatin in human ovarian cancer cell line C13K with multidrug-resistance by decreasing the expression of p-Akt. |
| | | | | 19067848 | Overexpression of P-AKT and NF-kappaB p65 were involved in the carcinogenesis and metastasis of ovarian cancer. |
| | | | | 22394200 | JNK-potentiated Akt/FoxO3a and JNK-mediated c-Jun pathways co-operatively trigger Puma expression, which determines the threshold for overcoming chemoresistance in ovarian cancer cells. |
| | | | | 23615713 | Results show that stromal cell-derived factor-1 (SDF-1) enhanced ovarian cancer cell invasion through alphavbeta6 integrin-mediated urokinase-type plasminogen activator (uPA) expression via the p38 MAPK and PI3 K/Akt pathway. |
| | | | | 25815442 | the results of the present study demonstrated that the acquired taxol resistance of ovarian cancer cells was associated with ROS-dependent upregulation in the expression of Tyro3 RTK and the subsequent activation of Akt. |
| | | | | 26364616 | gankyrin regulates HIF-1alpha protein stability and cyclin D1 expression, ultimately mediating FSH-driven ovarian cancer cell proliferation |
| | | | | 26713367 | Taken together, in current study, we found a novel tumor suppressor, DDX10, is epigenetic silenced by miR-155-5p in ovarian cancer, and the down-regulated expression pattern of DDX10 promotes ovarian cancer proliferation through Akt/NF-kappaB pathway. |
| | | | | 28423620 | High AKT1 expression is associated with ovarian cancer. |
| | | | | 28634229 | Data suggest that CAMKK2 is highly expressed in high-grade ovarian cancer and ovarian cancer cell lines; CAMKK2 directly activates Akt1 by phosphorylation at Thr-308 in a Ca2+/calmodulin-dependent manner; CAMKK2 knockdown or inhibition decreases Akt1 phosphorylation at Thr-308 and Ser-473. (CAMKK2 = calcium/calmodulin dependent protein kinase kinase 2; AKT1 = AKT serine/threonine kinase 1) |
| | | chr17:68697977-68699977<br>chr17:68734530-68736530 | SOX9 (6662) | 24661907 | Sox9 allows the survival of ovarian cells upon hypoxic condition, through the activation of betaIII-tubulin expression and its aberrant activation is prominent in patients with aggressive ovarian cancer. |
| ***16*** | ***pancreatic cancer*** | | | | |
| | | chr17:68734530-68736530<br>chr17:68746322-68748322 | SOX9 (6662) | 27599506 | Study demonstrated downregulation of expression of pancreatic master genes SOX9, FOXA2, and GATA4 (2-, 5-, and 4-fold, respectively) and in PANC1 pancreatic cancer cell line stimulated with TGFbeta1 |
| ***17*** | ***papillary thyroid carcinoma*** | | | | |
| | | chr17:32559802-32586800 | CCL2 (6347) | 19072670 | MCP-1 expression in papillary thyroid carcinoma may stimulate the aggressive behavior of this tumor or it may be a marker for aggressive behavior. |
| ***18*** | ***prostate cancer*** | | | | |
| | | chr1:173886202-173887200 | SERPINC1 (462) | 12466122 | Data show that anti-thrombin is widely expressed in prostate cancer but is gradually lost in tumors of high Gleason grade. |
| | | chr7:156583402-156584600 | SHH (6469) | 15314219 | Data report expression of sonic hedgehog-GLI-1 pathway components in adult human prostate cancer, often with enhanced levels in tumors versus normal prostatic epithelia. |
| | | | | 19254376 | Shh-expressing prostate cancer cells can directly and specifically induce differentiation in pre-osteoblasts. |
| | | | | 24925370 | Overexpression of SHH enhances prostate cancer cell lines resistance to paclitaxel. |
| ***19*** | ***rheumatoid arthritis*** | | | | |
| | | chr2:68643202-68648400 | PLEK (5341) | 26686060 | Only one gene, pleckstrin , was significantly overexpressed in periodontitis,cardiovascular disease, rheumatoid arthritis and ulcerative colitis , implicating this gene as an important networking link between these chronic inflammatory diseases |
| | | chr17:40504602-40508200 | STAT3 (6774) | 24708416 | IL-17 influences the innate immune system in rheumatoid arthritis by increasing the synovial expression of TLR3 via the STAT3 pathway. |
| | | | | 28987940 | Apoptosis in rheumatoid arthritis fibroblast-like synoviocytes is induced by targeting the expression of STAT3. |
| ***20*** | ***sarcoma*** | | | | |
| | | chr12:12002002-12021600 | ETV6 (2120) | 21457573 | highly expressed in human chondrosarcoma and promotes drug resistance in chondrosarcoma cells in vitro |
| ***21*** | ***tuberculosis*** | | | | |
| | | chr3:46409402-46417600 | CCR5 (1234) | 21592988 | The CCR5-HHD haplotype, a known genetic determinant of increased susceptibility to HIV-AIDS, and a high copy number of CCL3L1, a known genetic determinant of enhanced CCL3/CCL3L1 chemokine expression, each associated with presence of tuberculosis. |
| | | chr6:31534802-31548600 | TNF (7124) | 16133992 | During tuberculosis, predisposition of CD4 T-cell subsets to apoptosis may involve both low expression of Bcl-2 and excessive expression of tumor necrosis factor(TNF)-alpha. |
| | | | | 19091593 | Results reveal the suppressive effect of 1,25(OH)(2)D(3) on single cell expression of IFN-gamma and TNF-alpha by CD3+CD4+ and CD3+CD8+ T cells in pulmonary tuberculosis. |
| | | | | 23593415 | Gene expression of several TNF-alpha dependent apoptotic genes (TNFR1, TNFR2, FLICE, FLIPs) of peripheral blood cells from cohorts of individuals with active tuberculosis or potential exposure to tuberculosis, was investigated. |
| | | | | 25528189 | The expression of TNF-alpha and CXCL9 in blood samples stimulated with a bacterial antigen distinguishes active tuberculosis patients from latent disease carriers and healthy controls. |
| ***22*** | ***ulcerative colitis*** | | | | |
| | | chr2:68643202-68648400 | PLEK (5341) | 26686060 | Only one gene, pleckstrin , was significantly overexpressed in periodontitis,cardiovascular disease, rheumatoid arthritis and ulcerative colitis , implicating this gene as an important networking link between these chronic inflammatory diseases |
